# Production of pyruvic acid with *Candida glabrata* using self-fermenting spent yeast cell dry powder as a seed nitrogen source

**DOI:** 10.1186/s40643-022-00593-5

**Published:** 2022-10-17

**Authors:** Qiyuan Lu, Xiaoyu Shan, Weizhu Zeng, Jingwen Zhou

**Affiliations:** 1 Science Center for Future Foods, Jiangnan University, 1800 Lihu Road, Wuxi, 214122 Jiangsu China; 2 National Engineering Laboratory for Cereal Fermentation Technology, Jiangnan University, 1800 Lihu Road, Wuxi, 214122 Jiangsu China; 3 School of Biotechnology and Key Laboratory of Industrial Biotechnology, Ministry of Education, Jiangnan University, 1800 Lihu Road, Wuxi, 214122 Jiangsu China; 4 Jiangsu Provisional Research Center for Bioactive Product Processing Technology, Jiangnan University, 1800 Lihu Road, Wuxi, 214122 Jiangsu China

**Keywords:** Pyruvic acid, *Candida glabrata*, Spray drying, Spent yeast cell dry powder, Fermentation optimization

## Abstract

Pyruvic acid is an important organic acid and a key industrial raw material. It is widely used in the chemical, agricultural, and food fields. *Candida glabrata* is the preferred strain for pyruvic acid production. The waste yeast cell for pyruvic acid fermentation with *C. glabrata* are rich in protein, amino acid, nucleic acid, and vitamins, as potential and cost-effective nitrogen source raw material. In this study, the potential of *C. glabrata* to produce pyruvic acid using spent yeast cell dry powder was evaluated. When 30 g/L of spray-dried spent yeast cell powder was used as the seed nitrogen source, a high titer of pyruvic acid was obtained. The pyruvic acid production reached 63.4 g/L with a yield of 0.59 g/g in a 5 L bioreactor. After scale-up to a 50 L bioreactor using the fermented spent yeast cell dry powder as a seed nitrogen source, 65.1 g/L of pyruvic acid was harvested, with a yield of 0.61 g/g. This study proposes a promising

approach for increasing the pyruvic acid titer and reducing the costs.

**Graphical Abstract:**

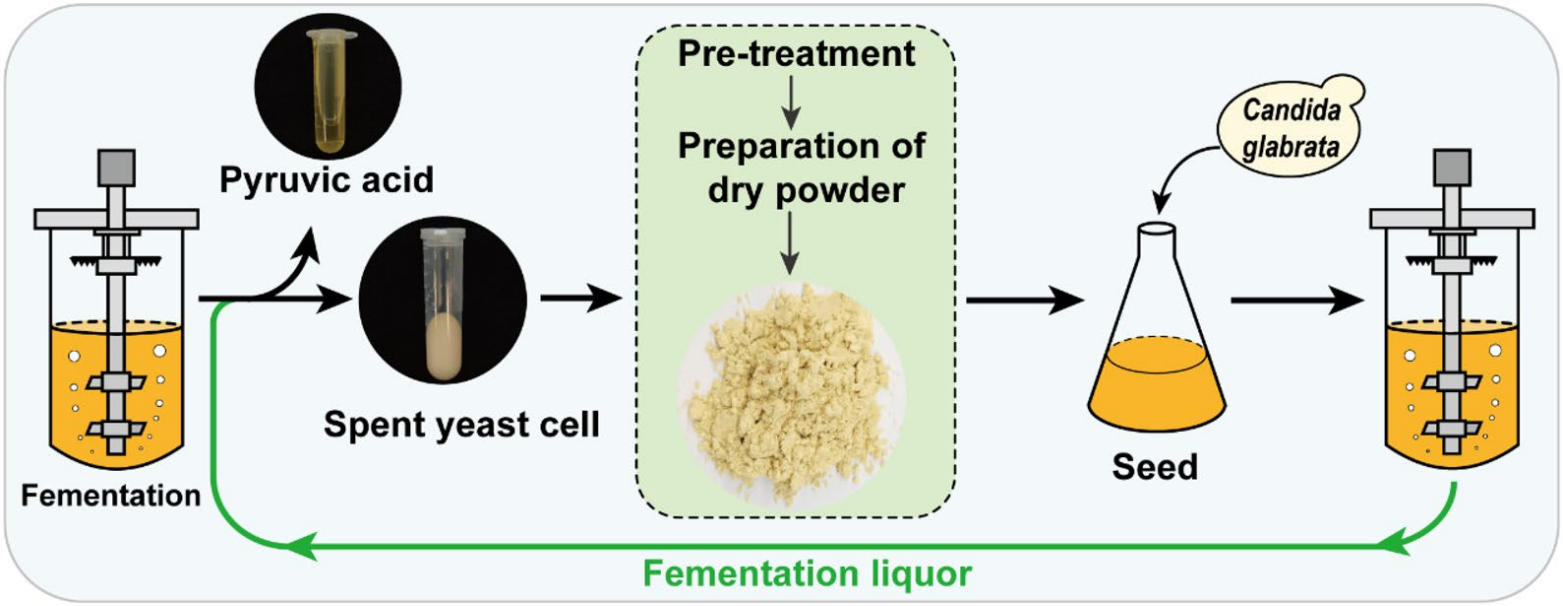

**Supplementary Information:**

The online version contains supplementary material available at 10.1186/s40643-022-00593-5.

## Introduction

Pyruvic acid is an important industrial raw material and it is widely used in various fields (Soma et al. 2022). In the agriculture field, pyruvic acid is often used to synthesize agrochemicals (Luo et al. 2020). In the food field, pyruvic acid can be used as a food additive (Yang and Xing. 2017). In the cosmetic field, pyruvic acid is used for anti-acne and anti-skin aging treatment (Anh et al. 2020). Pyruvic acid is also an important metabolic intermediate compound linking microbial nitrogen metabolism and carbon metabolism. Microbial cells can generate pyruvic acid from glucose by the glycolytic pathway, which is accompanied by the production of cofactors ATP and NADH. The pyruvic acid generated by glycolysis in eukaryotes mostly enters the mitochondria to participate in the TCA cycle to provide energy and a carbon source for cell growth (Maleki and Eiteman. 2017). In addition, pyruvic acid can undergo transamination to form alanine, which enters the nitrogen metabolism pathway of cells.

At present, the synthesis methods of pyruvic acid mainly include chemical synthesis, biotransformation, and microbial fermentation. Industrial pyruvic acid production methods mainly adopt the chemical synthesis approach. Here, tartaric acid was used as raw material, and the substrates tartaric acid and potassium pyrophosphate were mixed and heated so that tartaric acid was dehydrated and decarboxylated to form pyruvic acid. The main shortcomings of this method were high costs and serious environmental pollution (Luo et al. 2017b). Biotransformation method utilized the catalytic properties of enzymes, such as lactate oxidase and catalase in microorganisms to catalyze the conversion of lactic acid into pyruvic acid (Li et al. 2020). However, the raw materials for the biotransformation method were generally expensive and not suitable for a wide range of industrial applications. Microbial fermentation refers to the process of converting raw biomass materials, such as glucose or glycerol into pyruvic acid by directly using a series of enzymes of living microorganisms. Fermentation has obvious advantages, such as low cost, high product quality, and environmental friendliness (Luo et al. 2017a). The microorganisms commonly used to produce pyruvic acid by fermentation include *Candida glabrata* (Chen et al. 2018), *Corynebacterium glutamicum* (Kataoka et al. 2019), *Escherichia coli* (Liu and Cao. 2018), and *Saccharomyces cerevisiae* (Shachar et al. 1984). Generally, *C. glabrata* is considered to be the optimal strain for pyruvic acid production.

In previous study, many strategies were used to enhance the production of pyruvic acid with *C. glabrata*. 68.7 g/L of pyruvic acid was achieved in the fermenter by manipulating the activity of pyruvic acid dehydrogenase bypass, namely, decreasing the activity of pyruvic acid decarboxylase and increasing the activity of acetyl-CoA synthetase (Liu et al. 2004). By using a copper ion inducible promoter MT-1 to regulation of intracellular ATP concentration, a pyruvic acid titer of 67.4 g/L acid was achieved in the fermenter (Zhou et al. 2009). By introducing two different NADH reoxidation pathways to reduce the level of NADH and ATP in *C. glabrata*, the accumulation efficiency of pyruvic acid was increased by 21% (Qin et al. 2011). The pyruvic acid production was enhanced when the ATP futile cycle system was introduced to reduce the intracellular ATP content (Luo et al. 2019). A high titer pyruvic acid-producing mutant strain was obtained by combining ARTP-based random mutagenesis and high-throughput screening methods. This strain has a strong potential to produce pyruvic acid using soy peptone as a seed nitrogen source (Guo et al. 2020a; Luo et al. 2017b). At present, the titer, yield, and productivity of pyruvic acid by microbial fermentation have been well unified, but the cost of industrial production of pyruvic acid is still high. Researchers increasingly focus on reducing the cost of industrial pyruvic acid production.

Soy peptone is commonly used as seed nitrogen source to produce pyruvic acid in industry. However, the high price of soy peptone increases the industrial production cost. As an organism, yeast cells are rich in amino acids, vitamins, and trace elements, which can promote the growth and metabolism of microorganisms and improve production efficiency (Vukasinovic-Milic et al. 2007). In the present work, the glucose was used as the carbon source, and the spent yeast cell was dried and used as a seed liquid nitrogen source for pyruvic acid production, replacing soy peptone. The efficacy of spray drying spent yeast cell powder and soybean peptone as seed nitrogen source was assessed. Then, the concentration of the spent yeast cell dry powder was optimized. The culture and fermentation conditions were further optimized in a 5 L fermenter. Finally, pyruvic acid was produced scale-up in a 50 L fermenter with the goal of increasing the pyruvic acid titer and reducing the costs.

## Materials and methods

### Microorganisms

*C. glabrata* 4H2 is a multi-vitamin auxotrophic mutant (thiamine, biotin, pyridoxine, and nicotinic acid) pyruvic acid overproducer, was obtained from a wild-type strain *C. glabrata* CCTCC M202019 treated with ARTP- and EMS-based mutagenesis in our previous study (Guo et al. 2020b).

### Medium and culture conditions

In the medium for slant, seed cultures contained 30 g/L glucose, 10 g/L soy peptones, 0.5 g/L MgSO_4_·7H_2_O, and 1 g/L KH_2_PO_4_, and 20 g/L agar was added for solidification. Initial fermentation medium contained 120 g/L glucose, 0.8–2.0 g/L MgSO_4_·7H_2_O, 2 g/L KH_2_PO_4_, 3 g/L CH_3_COONa, 3.86 g/L urea, 10 mL/L trace element mixture, and 10–20 mL/L vitamin mixture. The initial pH of all cultures was adjusted to 5.5. The filter-sterilized trace element mixture and vitamin mixture were added to initial fermentation medium prior to inoculation. The trace element mixture contained 12 g/L MnCl_2_·4H_2_O, 2 g/L FeSO_4_·7H_2_O, 2 g/L CaCl_2_·2H_2_O, 0.05 g/L CuSO_4_·5H_2_O, and 0.5 g/L ZnCl_2_. The vitamin mixture contained 0.004 g/L biotin, 0.00075 g/L thiamine, 0.04 g/L pyridoxine, and 0.8 g/L niacin (Luo et al. 2018).

*C. glabrata* 4H2 was inoculated from an agar slant and incubated in seed culture medium in flasks. A seed culture was grown in flasks at 30 °C and 220 rpm overnight (around 16–18 h), collected, and 10% (*v/v*) of seed culture was inoculated into a 250 mL flask containing 25 mL of culture medium and incubated at 30 °C and 220 rpm on a reciprocal shaker (Zhichu, Shanghai, China). For flask culture, 40 g/L of CaCO_3_ was added to the medium prior to inoculation.

### Scale‑up biomass fermentation in a 5 L bioreactor and in a 50 L bioreactor

Batch fermentations were performed in a 5 L fermenter (Zhichu, Shanghai, China) with a 3 L working volume, and the pH was controlled automatically at 5.5 using 8 M NaOH. The agitation speed was 500 rpm, the aeration rate was 1.5 vvm (volumes of air per volume of broth per minute), the temperature was controlled at 30 °C, and the inoculation size was 10% (*v/v*). Based on batch fermentation process, the medium formula was optimized in a 5 L bioreactor. The initial fermentation medium was supplemented with different vitamin concentrations (10, 12.5, 15, 17.5, and 20 mL/L) and Mg^2+^ concentrations (0.8, 1.0, 1.2, 1.5, and 2.0 g/L) to produce pyruvic acid.

Based on the 5 L bioreactor fermentation process, scale-up was carried out in a 50 L bioreactor (Bailun, Shanghai, China) with a working volume of 30 L. A seed culture was inoculated into a 2000 mL flask containing 200 mL of culture medium and incubated at 30 °C and 220 rpm on a reciprocal shaker. Other parameters, such as temperature and pH, were taken with the same setup as 5 L fermenter. The bioreactor pressure was controlled at 0.035 MPa. Considering that the 50 L bioreactor controls pressure, the aeration rate of 1.5 vvm was too high, easily leading to an increase in foam, so it was reduced to 1.0 vvm. At the same time, mechanical stirring and shearing at a high rotational speed was not conducive to the growth of cells, so after inoculation, the rotation speed and aeration rate increased step by step according to the following operation: 0–4 h, 200 rpm, 10 L/min; 4–8 h, 300 rpm, 20 L/min; 8–60 h, 400 rpm, 30 L/min; and 60–95 h, 500 rpm, 30 L/min.

### Preparation of spent yeast cell dry powder

The fermentation broth was centrifuged for 30 min, 3500 rpm (Beckman, Los Angeles, USA), and the supernatant was removed to obtain the solid. The solid was resuspended with deionized water and centrifuged again for 15 min to obtain the solid. And the solid was dried and used as the nitrogen source of the seed medium for the next batch of pyruvic acid production. Three different methods were used to dry spent yeast cells: (i) For freeze drying, the spent yeast cells were pre-frozen in a − 80 °C freezer for 4 h and transferred to a freeze dryer for 20 h. (ii) For hot-air drying, the spent yeast cells were dried with hot air at a constant temperature (65 °C) for 24 h, and the spent yeast cells were stirred every 8 h. (iii) For spray drying, spent yeast cells were contacted with hot air in a dryer, and the moisture was evaporated instantly to obtain a dry yeast powder. The inlet air temperature of the drying tower was 130 °C and the exhaust air temperature was 80 °C.

### Analytical method

Taking an appropriate volume of fermentation broth, an appropriate amount of HCl was added to remove CaCO_3_, and then deionized water was added to dilute the fermentation broth for measuring OD_660_. The dry cell weight (DCW) was calculated as follows: DCW = 0.23 × OD_660_ (Guo et al. 2020b). The glucose content in the fermentation broth was determined by a glucose-lactic acid biosensor analyzer (Sieman Technology, Shenzhen, China). The pyruvic acid content was detected and analyzed by high-performance liquid chromatography (Shimadzu, Kyoto, Japan). The chromatographic detection conditions were as follows: the column was an ion exchange column (Bio Rad Aminex HPX-87H column, USA), the detection wavelength was 210 nm, the temperature of the column oven was 40 °C, the liquid input volume was 10 μL, the flow rate was 0.5 mL/min, and the mobile phase was 5 mM dilute H_2_SO_4_. The samples were diluted as appropriate and subjected to filtration using a 0.22 μm membrane (Luo et al. 2020).

## Results and discussion

### Effects of different nitrogen sources and different spent yeast cell dry powders on pyruvic acid production

**Fig. 1:**
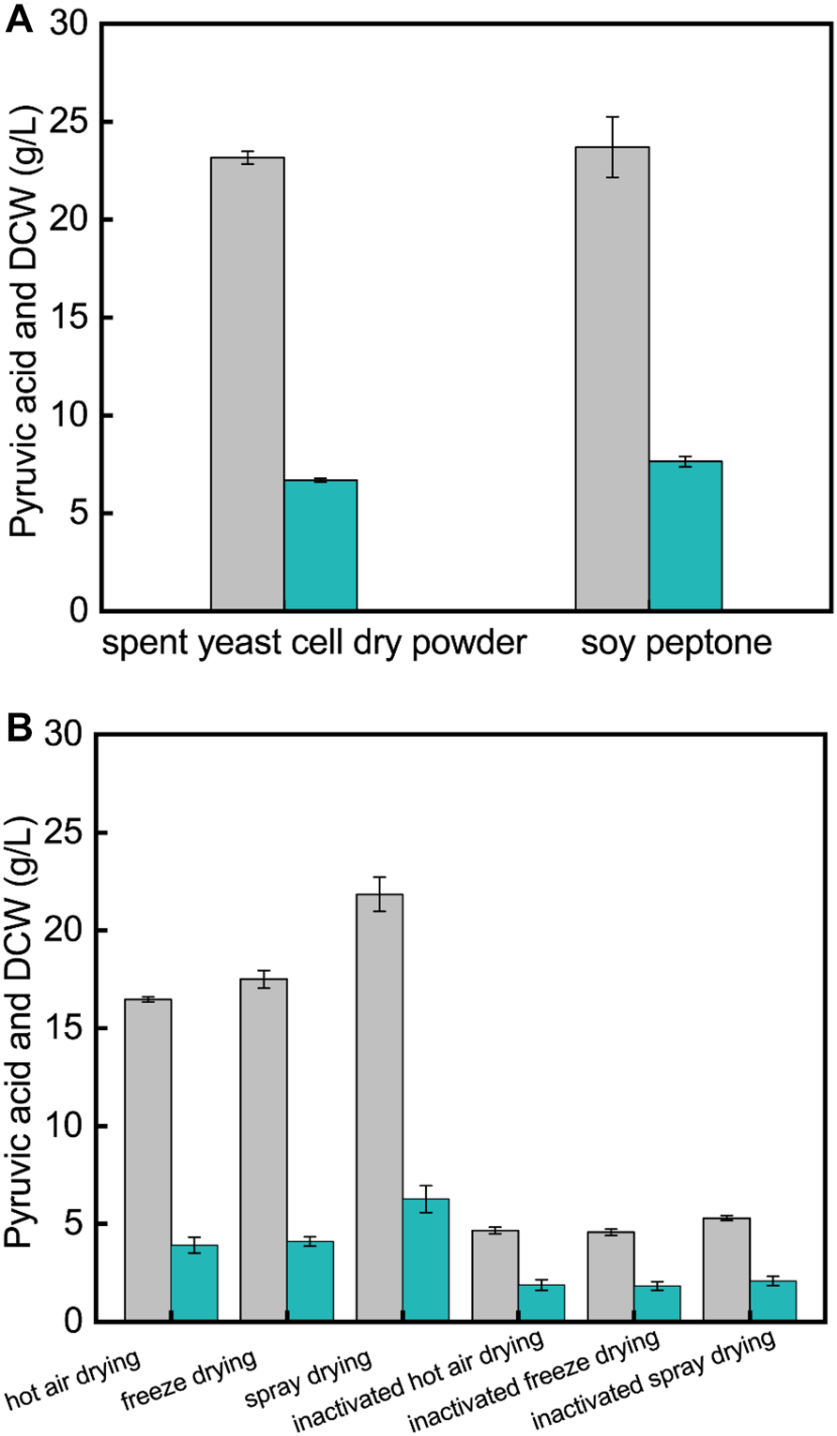
Production of pyruvic acid with different nitrogen sources and different spent yeast cell dry powders. **A**: Effects of nitrogen sources on cell growth and acid production. **B**: Effects of different spent yeast cell dry powders on cell growth and acid production. Gray columns, pyruvic acid; blue columns, DCW

In the fermentation process of pyruvic acid production with *C. glabrata*, nitrogen is the most important component in the seed medium, and its type has an important influence on the growth of cell in seeds. The vigor of the cells in the seed solution could affect the fermentation results to a large extent. Seeds with strong vigor are conducive to rapid growth of the cells, shorten the lag period, and maintain a high acid production capacity (Kenan. 1984). In a pre-experiment, effects of soy peptone and spent yeast cell dry powder with same concentration (10 g/L) on cell growth in seed medium were investigated. Trend of cell growth with spent yeast cell dry powder was basically consistent with that of with the soy peptone, but the biomass was slightly lower than that of with the soy peptone (data not shown). Effects of two different nitrogen sources (10 g/L) on cell growth and pyruvic acid accumulation in the fermentation process were further compared. The results are shown in Fig. 1A. When soy peptone was used as nitrogen source in seed medium, the DCW was 7.6 g/L and a pyruvic acid titer of 23.7 g/L was achieved, while when the spray-dried spent yeast cell powder was used as nitrogen source in seed medium, the DCW reached 6.7 and a pyruvic acid titer of 23.2 g/L was achieved. Both nitrogen sources could meet the requirements of cell growth and acid production. Therefore, spent yeast cell dry powder was a suitable nitrogen source as well.

Different processing methods of spent yeast cell dry powder have different effects on the nutrients. The effects of spent yeast cell dry powder obtained with different drying processing methods on cell growth and acid production were further investigated. The same concentrations of various spent yeast cell dry powders were used as nitrogen sources. The results of shake flask fermentation are shown in Fig. 1B. When the spray-dried spent yeast cell powder was used as nitrogen source, the pyruvic acid titer was 21.9 g/L and the DCW was 6.3 g/L. The production of pyruvic acid was 32.73% and 25.14% higher than those obtained with hot-air drying and freeze drying, respectively, and the DCW was also increased by 61.54% and 53.66%, respectively. Besides, when drying the cells after inactivation, both the pyruvic acid accumulation and the cell concentration were significantly lower than those before inactivation. The results indicated that drying before inactivation of the cells was favorable for cell growth and pyruvic acid accumulation.

Pyruvic acid has shown promise in the beverage and food industries, as well as the chemical, agricultural fields. The industrial production of pyruvic acid is essential to meet the growing demand. When pyruvic acid is produced by microbial fermentation, the microbial cells are often discarded as waste at the end of fermentation, resulting in environmental pollution and resource waste. However, microbial cells used in the fermentation industry contain proteins, vitamins, trace elements, and other substances, which are nutrient-rich resources (Koike and Gordon. 2015). Therefore, it is of great significance to recycle and utilize spent microbial cells as raw materials to explore and develop by-products with higher value (Wu et al. 2016). The dry matter of spent cells contains about 50% protein, which has great potential for use as organic nitrogen sources, but currently there are few research reports, mainly focusing on the development of biological fertilizers and condiments (Zhang et al. 2021). A study showed that the hydrolysate of *Candida* cells when used as a nitrogen source can replace 50% of yeast extract for the production of *α*, *ω*-dodecanedioic acid (Cao et al. 2018). Besides, spent yeast cell hydrolysate can enhance the succinic acid concentration when used as a nitrogen source (Chen et al. 2011). In the fermentation process of pyruvic acid production with *C. glabrata*, the spent yeast cells after fermentation are also rich in protein, amino acids, vitamins, and other substances, so spent yeast cell dry powder could also replace organic nitrogen sources to produce pyruvic acid.

### Effects of different concentrations of spray-dried spent yeast cell powder on pyruvic acid accumulation

**Fig. 2:**
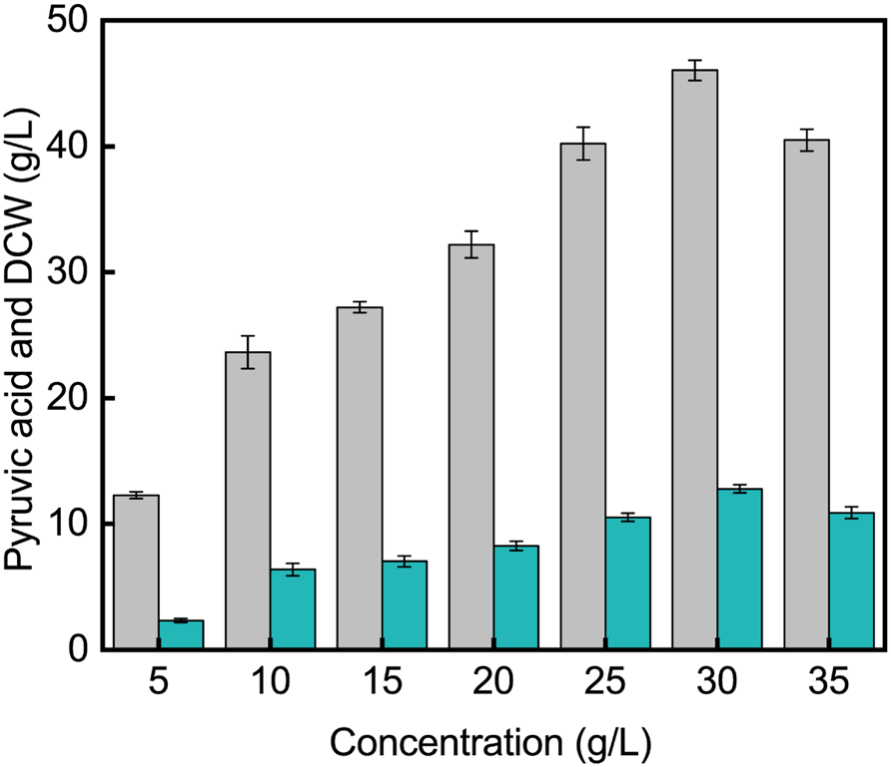
Effect of different concentrations of spray-dried spent yeast cell powder on pyruvic acid accumulation. When the concentration of spray-dried spent yeast cell powder was below 30 g/L, the pyruvic acid titer and the biomass increased with increasing concentration of spray-dried spent yeast cell powder. When the concentration was 35 g/L, the pyruvic acid titer and the biomass decreased. Gray columns, pyruvic acid; blue columns, DCW

After the spray-dried spent yeast cell powder was determined to be optimal for cell growth and pyruvic acid accumulation, the effects of different concentrations of spent yeast cell dry powder (5, 10, 15, 20, 25, 30, and 35 g/L) on the pyruvic acid fermentation process were further investigated. The pyruvic acid titer and the biomass increased with increasing concentrations of spray-dried spent yeast cell powder as long as the concentration remained below 30 g/L. When the concentration of spray-dried spent yeast cell powder was 30 g/L, the pyruvic acid titer and the biomass were highest, reaching 46.1 g/L and 12.8 g/L, respectively. When the cell dry powder concentration was further increased, the pyruvic acid titer and the biomass decreased (Fig. 2).

The nitrogen types and ingredients of nitrogen sources could affect the growth of cells and acid production. Suitable nitrogen sources could improve production efficiency and reduce production costs. For example, inorganic nitrogen sources are conducive to riboflavin production and organic nitrogen sources are conducive to the accumulation of antifungal components (Kim et al. 1981; Zotkina and Koroleva. 1984). Generally, organic nitrogen sources are expensive, which increases the fermentation cost. A common strategy is to use cheap alternative nitrogen sources to reduce costs (Li et al. 2021). In this study, spent yeast cell dry powder was used as nitrogen source instead of soy peptone to produce pyruvic acid. The market price of soy peptone was relative higher, while the spent yeast cells could be harvested by a simple and less investment drying process. A portion of the obtained spent yeast cell dry powder was applied as nitrogen source to reduce the cost of pyruvic acid production. In addition, the most rest of spent yeast cell dry powder could also be used to prepare biofertilizer and feeds, bringing in additional benefits (Ferreira et al. 2010).

### Comparison between the spray-dried spent yeast cell powder and soy peptone in a fermenter

**Fig. 3:**
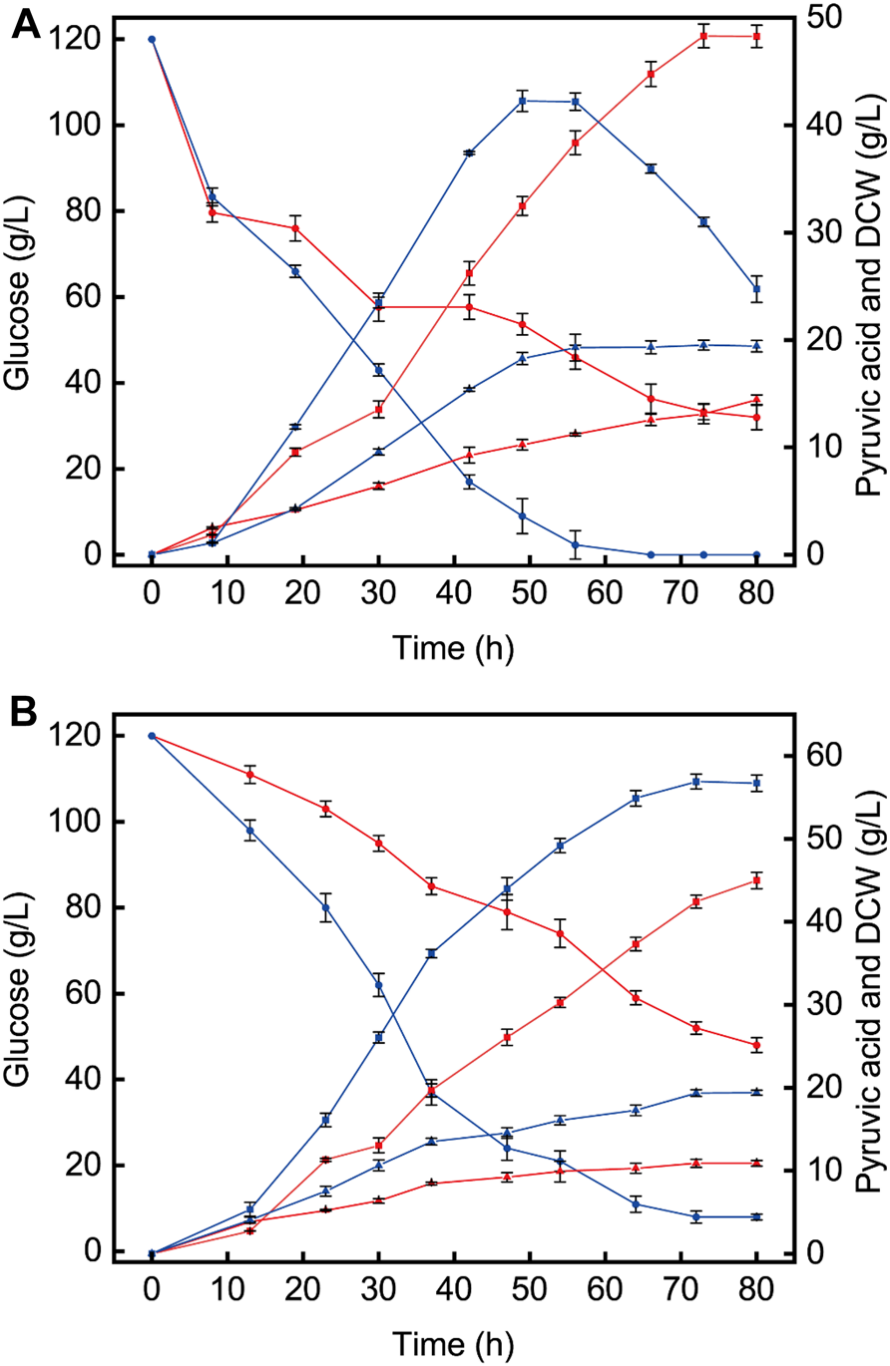
Comparison between spray-dried spent yeast cell powder and soy peptone. **A**: Time courses of batch fermentation in shaking flasks. **B**: Time courses of batch fermentation in a fermenter. Blue lines, soybean peptone; red lines, spray-dried spent yeast cell powder; triangles, DCW; circles, glucose; squares, pyruvic acid

In the previous study, it has been proved that the optimal concentration of soy peptone was 10 g/L (Guo et al. 2020b). Based on the above result that addition of 30 g/L spray-dried spent yeast cell powder was beneficial to pyruvic acid accumulation; therefore, comparative experiments were conducted with the initial addition of soy peptone (10 g/L) in shaking flasks. Compared with soy peptone as nitrogen source, the accumulation of pyruvic acid increased when using 30 g/L spray-dried spent yeast cell powder, reaching 48.3 g/L, while the highest value with soy peptone was 42.2 g/L. But the biomass with the spray-dried spent yeast cell powder was lower, glucose consumption was relatively slow, and there was some residue at the end of the fermentation process (Fig. 3A). To investigate the specific effects of spray-dried spent yeast cell powder, the comparison was further carried out in a 5 L fermenter. The effect of the spray-dried spent yeast cell powder was disappointing, which differed from that in shaking flasks. The pyruvic acid titer was only 45.0 g/L with the spray-dried spent yeast cell powder as nitrogen source for seeds, while the pyruvic acid production could reach 56.9 g/L with soy peptone. Besides, the biomass with the spray-dried spent yeast cell powder (10.9 g/L) was also obviously lower than that obtained with soy peptone (19.7 g/L) (Fig. 3B).

The composition and proportion of the culture medium have an important impact on microbial cell growth and product synthesis (Peter et al. 2021). Previous reports showed that the production of aromatic compounds by nonconventional yeast fermentation was improved by adding tequila vinasses into the medium (Rodriguez-Romero et al. 2020). The concentration of some metal ions in the medium also had a significant effect on the production of fermentation metabolites (Muneeswaran et al. 2021). In this study, in spite of using 30 g/L of spent yeast cell dry powder in both shake flask and fermenter, the lower pyruvic acid obtained in fermenter compared to shake flask was disappointing. It was speculated that factors such as insufficient addition of some important components in the medium and differences in fermentation process. There are differences in dissolved oxygen levels and mechanical injury between shake flask fermentation and fermenter fermentation. Dissolved oxygen levels and shear forces from excessive agitation affect cell growth and product synthesis (Flores-Copa et al. 2021; Snopek et al. 2021).

### Optimization of the medium formula to promote the pyruvic acid production

**Fig. 4:**
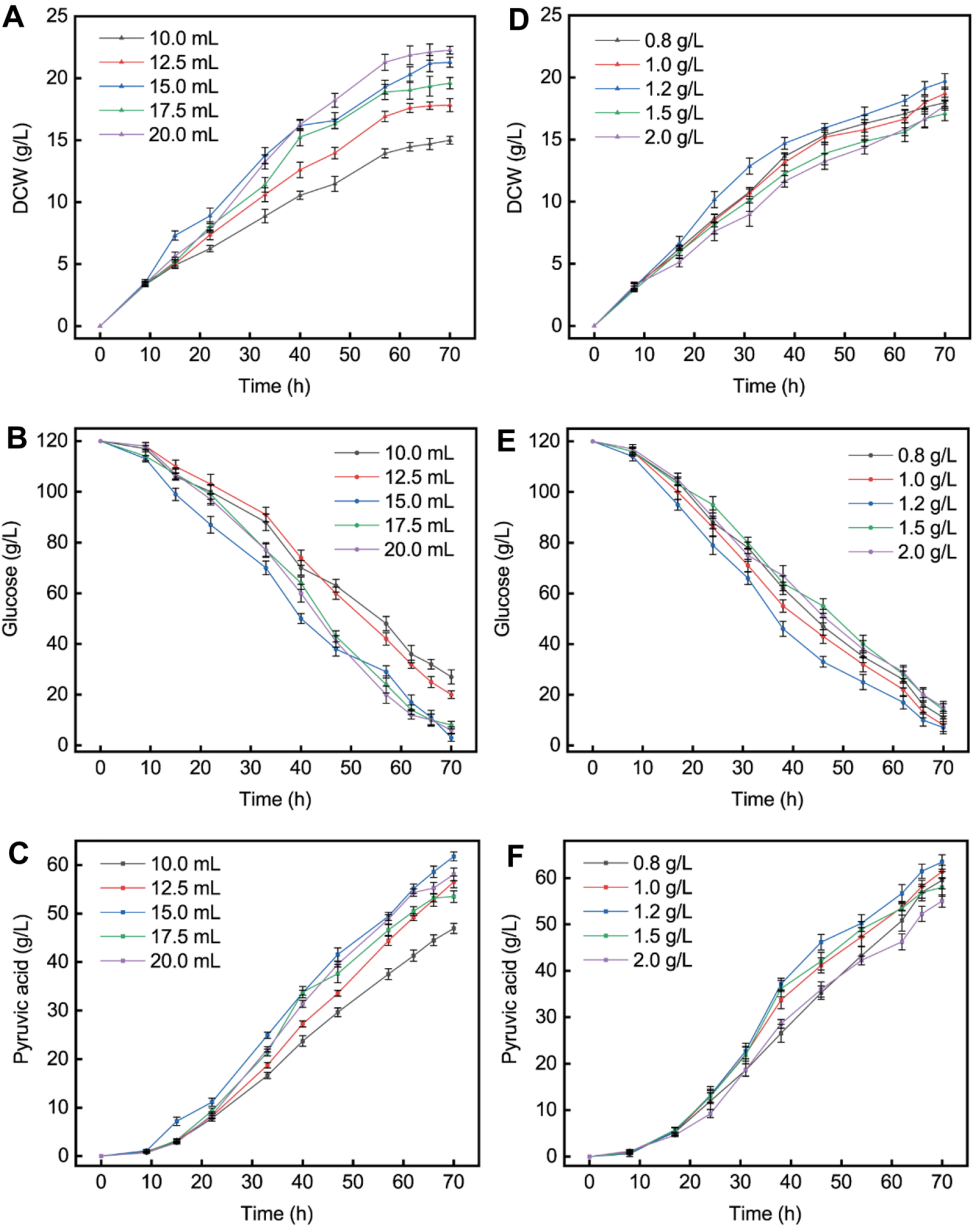
Fermentation medium optimization in a 5 L bioreactor. **A** Effect of vitamin addition on cell growth. **B** Effect of vitamin addition on glucose consumption. **C** Effect of vitamin addition on pyruvic acid synthesis. **D** Effect of Mg^2+^ concentration on cell growth. **E** Effect of Mg^2+^ concentration on glucose consumption. **F** Effect of Mg^2+^ concentration on pyruvic acid biosynthesis. Triangles, DCW; circles, glucose; squares, pyruvic acid

The spray-dried spent yeast cell powder as nitrogen source in seed medium to produce pyruvic acid in fermenter was not ideal. The contents of some components in the fermentation medium might be insufficient, so it was necessary to optimize the fermentation medium. *C. glabrata* 4H2 is a four-vitamin auxotrophic strain, and the effect of mixture concentration of four vitamins on pyruvic acid production was studied on the basis of 30 g/L spray-dried spent yeast cell powder as seed nitrogen source (Fig. 4A–C and Table 1)*.* A significant and sustained improvement in cell growth was observed with increasing amounts of vitamin. The maximum biomass (21.3 g/L) was obtained with 20 mL/L of vitamin addition, but the highest pyruvic acid titer (61.7 g/L) was obtained with 15 mL/L of vitamin addition. When 10 mL/L, 12.5 mL/L, or 17.5 mL/L vitamins was added, the glucose was not fully utilized (the utilization rates were 77.5, 83.3, and 93.3%, respectively). The rate of glucose consumption was significantly lower at low vitamin levels (10 mL/L or 12.5 mL/L addition) than at high vitamin levels (15 mL/L, 17.5 mL/L, or 20 mL/L addition). At the end of fermentation, the remaining amounts of four vitamins were detected (Additional file 1: Fig. S1 and Additional file 1: Table S1). These results showed that the fermentation effect was best with 15 mL/L of vitamin addition.

**Table 1:** Effects of vitamin addition on the production of pyruvic acid by *C. glabrata*

Addition (mL/L)	DCW (g/L)	Pyruvic acid (g/L)	Yield (g/g)	productivity (g/L/h)
10.0	15.0	46.9	0.53	0.67
12.5	17.8	56.6	0.59	0.81
15.0	21.3	61.7	0.55	0.88
17.5	19.6	53.5	0.50	0.76
20.0	22.3	58.1	0.53	0.83

In addition, Mg^2+^ is a cofactor for many enzymatic reactions. It is involved in the synthesis of proteins and nucleic acids and stabilizes and protects biological membranes. The effect of Mg^2+^ on cell growth and pyruvic acid accumulation was investigated. The results are presented in Fig. 4D–F and Table 2. When the Mg^2+^ concentration in the medium was 1.2 g/L, the biomass (19.7 g/L) and pyruvic acid production (63.4 g/L) were highest. A conversion rate of 0.59 g/g and a productivity of 0.91 g/L/h were achieved.

**Table 2:** Effect of Mg^2+^ on the production of pyruvic acid by *C. glabrata*

Addition (g/L)	DCW (g/L)	Pyruvic acid (g/L)	Yield (g/g)	productivity (g/L/h)
0.8	18.0	59.5	0.57	0.85
1.0	18.7	61.4	0.58	0.88
1.2	19.7	63.4	0.59	0.91
1.5	17.1	58.0	0.57	0.83
2.0	17.7	55.0	0.55	0.79

The concentration of vitamin plays a key role in cell growth and pyruvic acid accumulation for vitamin auxotrophic strains (Sprenger et al. 2020; Walker et al. 2020). By optimizing the thiamine concentration, the titer of pyruvic acid produced by *Yarrowia lipolytica* was significantly increased (Cybulski et al. 2019). With the addition of the optimal concentration of vitamin, the yield of pyruvic acid synthesis by *C. glabrata* increased (Li et al. 2001). These studies clearly demonstrated the importance of vitamins. In addition, Mg^2+^ plays a role in enhancing oleaginous yeast, which has been confirmed to accumulate palmitoleic acid (Zhou et al. 2021). It has been reported that biomass, glucose consumption, and pyruvic acid accumulation increase with increasing Mg^2+^ levels over a range of concentrations. However, a high Mg^2+^ concentration cannot effectively improve the consumption of glucose and the titer of pyruvic acid, which is consistent with present results (Venuti et al. 1989).

### Scale-up of pyruvic acid production in a 50 L fermenter

**Fig. 5:**
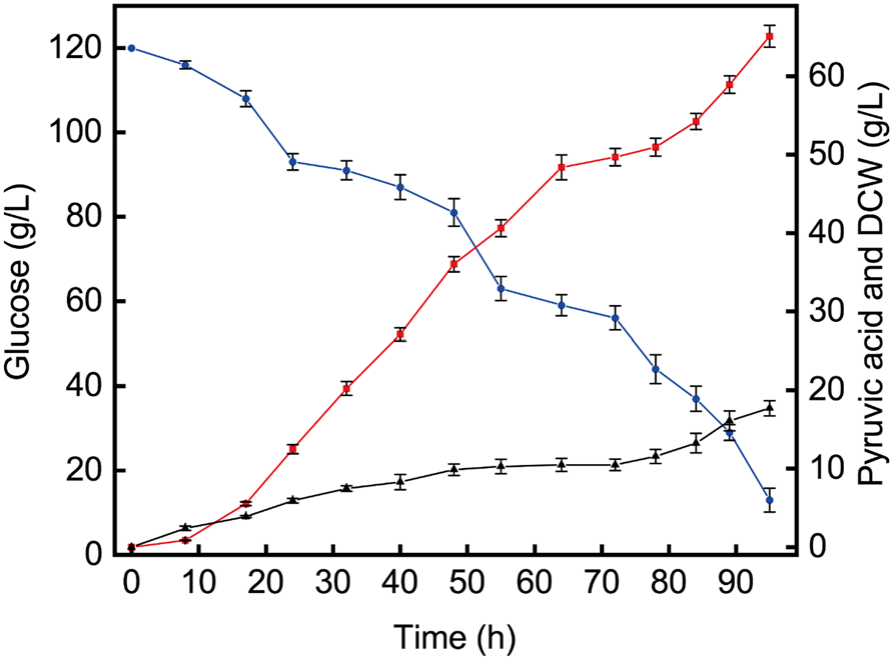
Time course of pyruvic acid biosynthesis in a 50 L bioreactor. The pressure of the fermenter was controlled at 0.035 MPa, and the stirring speed and aeration were amplified step by step (0–4 h, 200 rpm, 10 L/min; 4–8 h, 300 rpm, 20 L/min; 8–60 h, 400 rpm, 30 L/min; 60–95 h, 500 rpm, 30 L/min). At the end of fermentation, the pyruvic acid titer was 65.1 g/L, and the conversion rate was 0.61 g/g. Triangles, DCW; circles, glucose; squares, pyruvic acid

In order to achieve the goal of industrialized production of pyruvic acid by fermentation of *C. glabrata*, a scale-up experiment with a 50 L bioreactor was carried out based on the 5 L bioreactor. The pressure of the fermenter was controlled at 0.035 MPa, and the stirring speed and aeration were amplified step by step. The results are shown in Fig. 5. It was found that before 64 h in the fermentation process, the accumulation of pyruvic acid increased rapidly, but it decreased after 64 h, and more glucose remained. When increasing the stirring speed at 72 h, the cells continued to grow, the glucose was rapidly consumed, and the accumulation of pyruvic acid was accelerated. At the end of fermentation, the dry weight of cells reached 17.7 g/L, the accumulation of pyruvic acid was 65.1 g/L, and the conversion ratio was 0.61 g/g. Compared with the 5 L bioreactor, the titer and the yield increased and the productivity decreased (Table 3).

**Table 3:** Comparison of different seed nitrogen sources

Fermentation parameters	Seed nitrogen sources				
	Soy peptone by flask	Spent yeast cell dry powder by shaking flask	Soy peptone by 5 L bioreactor	Spent yeast cell dry powder by 5 L bioreactor	Spent yeast cell dry powder by 50 L bioreactor
Fermentation period (h)	56	72	72	70	95
Glucose consumption (g/L)	117	87	112	113	107
Pyruvic acid production (g/L)	42.2	48.3	56.9	63.4	65.1
Yield of pyruvic acids on glucose (g/g)	0.36	0.55	0.53	0.59	0.61
Pyruvic acids productivity (g/L/h)	0.75	0.67	0.79	0.91	0.69

The scale-up experiment in a 50 L bioreactor suggested that it was possible to replace soy peptone with spray-dried spent yeast cell powder for industrial production of pyruvic acid. In the future, some fermentation parameters, such as temperature, pH, and dissolved oxygen levels, could be optimized to enhance the production of pyruvic acid and increase the benefits of enterprises (Chidi et al. 2018; Kenan. 1984). In addition, fed-batch strategies have developed into proven methods to increase the production of certain products, such as 2-phenylethanol, L-tyrosine, and keto acids (Lei et al. 2019; Tian et al. 2020; Zeng et al. 2017). A previous study has shown that a high pyruvic acid titer could be obtained with *C. glabrata* with a lower initial glucose concentration and a fed-batch strategy (Debruin. 1988). The feed optimization strategies can also be further studied in combination with other methods to increase pyruvic acid accumulation, such as analysis of tail gas and kinetic parameters of fermentation processes. The production of pyruvic acid was enhanced significantly by using a fed-batch fermentation strategy based on analysis of fermentation kinetic parameters (Guo et al. 2020b). Moreover, the synthesis of pyruvic acid can be further improved by feeding spray-dried powder to promote the secondary growth of cells.

## Conclusion

Medium cost is a key factor in the fermentative production of biochemicals. The use of cheap raw materials instead of soy peptone is important for the economic production of pyruvic acid. In this study, spray-dried spent yeast cell powder was used to replace soy peptone as the seed nitrogen source for producing pyruvic acid with *C. glabrata*. There was no significant difference in the production of pyruvic acid between the two nitrogen sources. By optimizing the concentration of spray-dried spent yeast cell powder and the composition of the growth medium, the pyruvic acid titer reached 63.4 g/L with a yield of 0.59 g/g in a 5 L bioreactor. Compared with soy peptone, the spray-dried spent yeast cell powder increased the titer of pyruvic acid by 11.4% in 5 L fermenters. Finally, scaling up in a 50 L fermenter, 65.1 g/L of pyruvic acid was obtained, with a yield of 0.61 g/g. The results showed that spray-dried spent yeast cell powder as a seed nitrogen source for pyruvic acid production had industrial potential.

### Supplementary Information

**Additional file 1: Figure S1.** Detection the remaining amounts of four vitamins. **Table S1.** The remaining amounts of four vitamins at the end of fermentation

## Data Availability

All data and materials are available as described in the research article and its supporting information document, which will be given access on the journal’s website.

## References

[CR1] Anh HTL, Kawata Y, Tam LT, Thom LT, Ha NC, Hien HTM, Thu NTH, Huy PQ, Hong DD (2020). Production of pyruvate from Ulva reticulata using the alkaliphilic, halophilic bacterium *Halomonas sp*. BL6. J Appl Phycol.

[CR2] Cao W, Wang Y, Luo J, Yin J, Wan Y (2018). Improving alpha, omega-dodecanedioic acid productivity from n-dodecane and hydrolysate of *Candida* cells by membrane integrated repeated batch fermentation. Bioresour Technol.

[CR3] Chen KQ, Li J, Ma JF, Jiang M, Wei P, Liu ZM, Ying HJ (2011). Succinic acid production by *Actinobacillus succinogenes* using hydrolysates of spent yeast cells and corn fiber. Bioresour Technol.

[CR4] Chen X, Luo Q, Liu J, Liu L (2018). Enhancement of pyruvate productivity in *Candida glabrata* by deleting the CgADE13 Gene to improve acid tolerance. Biotechnol Bioprocess Eng.

[CR5] Chidi BS, Bauer FF, Rossouw D (2018). the impact of changes in environmental conditions on organic acid production by commercial wine yeast strains. S Afr J Enol Vitic.

[CR6] Cybulski K, Tomaszewska-Hetman L, Rakicka M, Juszczyk P, Rywinska A (2019). Production of pyruvic acid from glycerol by *Yarrowia lipolytica*. Folia Microbiol.

[CR7] Debruin TW (1988). Difference in species specificity of TSH receptor antibodies in graves' disease and hashimoto's thyroiditis. J Endocrinol Invest.

[CR8] Ferreira I, Pinho O, Vieira E, Tavarela JG (2010). Brewer's saccharomyces yeast biomass: characteristics and potential applications. Trends Food Sci Technol.

[CR9] Flores-Copa V, Romero-Soto L, Romero-Calle D, Alvarez-Aliaga MT, Orozco-Gutierrez F, Vega-Baudrit J, Martin C, Carrasco C (2021). Residual brewing yeast as substrate for Co-production of cell biomass and biofilm using *Candida maltosa SM4*. Fermentation-Basel.

[CR10] Guo L, Zeng W, Xu S, Zhou J (2020). Fluorescence-activated droplet sorting for enhanced pyruvic acid accumulation by *Candida glabrata*. Bioresour Technol.

[CR11] Guo L, Zeng W, Zhou J. (2020b) Process optimization of fed-batch fermentation for pyruvic acid production with *Candida glabrata*. Food and Fermentation Industries 46:10–16.

[CR12] Kataoka N, Vangnai AS, Pongtharangkul T, Yakushi T, Wada M, Yokota A, Matsushita K (2019). Engineering of *Corynebacterium glutamicum* as a prototrophic pyruvate-producing strain: characterization of a ramA-deficient mutant and its application for metabolic engineering. Biosci Biotechnol Biochem.

[CR13] Kenan PD (1984). Management of nose bleed. N C Med J.

[CR14] Kim HC, Kemmann E, Shelden RM, Saidi P (1981). Response of blood coagulation parameters to elevated endogenous 17 beta-estradiol levels induced by human menopausal gonadotropins. Am J Obstet Gynecol.

[CR15] Koike ST, Gordon TR (2015). Management of *Fusarium* wilt of strawberry. Crop Prot.

[CR16] Lei Q, Zeng W, Zhou J, Du G (2019). Efficient separation of alpha-ketoglutarate from *Yarrowia lipolytica* WSH-Z06 culture broth by converting pyruvate to l-tyrosine. Bioresour Technol.

[CR17] Li Y, Chen J, Lun SY, Rui XS (2001). Efficient pyruvate production by a multi-vitamin auxotroph of *Torulopsis glabrata*: key role and optimization of vitamin levels. Appl Microbiol Biotechnol.

[CR18] Li GS, Lian JZ, Xue HL, Jiang YQ, Wu MB, Lin JP, Yang LR (2020). Enzymatic preparation of pyruvate by a whole-cell biocatalyst coexpressing L-lactate oxidase and catalase. Process Biochem.

[CR19] Li C, Zhai X, Wen Q, Wang S. (2021) Effect of feeding different organic nitrogen sources on L-lysine fermentation. Cereal & Food Industry 28:44–47

[CR20] Liu LM, Cao ZJ (2018). Regulation of NADH oxidase expression via a thermo-regulated genetic switch for pyruvate production in *Escherichia coli*. Biotechnol Bioprocess Eng.

[CR21] Liu LM, Li Y, Li HZ, Chen J (2004). Manipulating the pyruvate dehydrogenase bypass of a multi-vitamin auxotrophic yeast *Torulopsis glabrata* enhanced pyruvate production. Lett Appl Microbiol.

[CR22] Luo Z, Liu S, Du G, Zhou J, Chen J (2017). Identification of a polysaccharide produced by the pyruvate overproducer *Candida glabrata* CCTCC M202019. Appl Microbiol Biotechnol.

[CR23] Luo Z, Zeng W, Du G, Liu S, Fang F, Zhou J, Chen J (2017). A high-throughput screening procedure for enhancing pyruvate production in *Candida glabrata* by random mutagenesis. Bioprocess Biosyst Eng.

[CR24] Luo Z, Liu S, Du G, Xu S, Zhou J, Chen J (2018). Enhanced pyruvate production in *Candida glabrata* by carrier engineering. Biotechnol Bioeng.

[CR25] Luo Z, Zeng W, Du G, Chen J, Zhou J (2019). Enhanced pyruvate production in *Candida glabrata* by engineering ATP futile cycle system. ACS Synth Biol.

[CR26] Luo Z, Zeng W, Du G, Chen J, Zhou J (2020). Enhancement of pyruvic acid production in *Candida glabrata* by engineering hypoxia-inducible factor 1. Bioresour Technol.

[CR27] Maleki N, Eiteman MA (2017). Recent progress in the microbial production of pyruvic acid. Fermentation-Basel.

[CR28] Muneeswaran G, Patel SKS, Kondaveeti S, Shanmugam R, Gopinath K, Kumar V, Kim SY, Lee JK, Kalia VC, Kim IW (2021). Biotin and Zn(2+) Increase xylitol production by *Candida tropicalis*. Indian J Microbiol.

[CR29] Peter AP, Chew KW, Koyande AK, Yuk-Heng S, Ting HY, Rajendran S, Munawaroh HSH, Yoo CK, Show PL (2021). Cultivation of *Chlorella vulgaris* on dairy waste using vision imaging for biomass growth monitoring. Bioresour Technol.

[CR30] Qin Y, Johnson CH, Liu LM, Chen JA (2011). Introduction of heterogeneous NADH reoxidation pathways into *Torulopsis glabrata* significantly increases pyruvate production efficiency. Korean J Chem Eng.

[CR31] Rodriguez-Romero JJ, Aceves-Lara CA, Silva CF, Gschaedler A, Amaya-Delgado L, Arrizon J (2020). 2-Phenylethanol and 2-phenylethylacetate production by nonconventional yeasts using tequila vinasses as a substrate. Biotechnol Rep (amst).

[CR32] Shachar E, Barzilay Z, Shohet I, Cohen BE (1984). Rifampin in osteoarthritis due to *Brucella melitensis*. Harefuah.

[CR33] Snopek P, Nowak D, Zieniuk B, Fabiszewska A (2021). Aeration and Stirring in *Yarrowia lipolytica* lipase biosynthesis during batch cultures with waste fish oil as a carbon source. Fermentation-Basel.

[CR34] Soma Y, Yamaji T, Hanai T (2022). Dynamic metabolic engineering of *Escherichia coli* improves fermentation for the production of pyruvate and its derivatives. J Biosci Bioeng.

[CR35] Sprenger M, Hartung TS, Allert S, Wisgott S, Niemiec MJ, Graf K, Jacobsen ID, Kasper L, Hube B (2020). Fungal biotin homeostasis is essential for immune evasion after macrophage phagocytosis and virulence. Cell Microbiol.

[CR36] Tian S, Liang X, Chen J, Zeng W, Zhou J, Du G (2020). Enhancement of 2-phenylethanol production by a wild-type *Wickerhamomyces anomalus* strain isolated from rice wine. Bioresour Technol.

[CR37] Venuti MC, Young JM, Maloney PJ, Johnson D, McGreevy K (1989). Synthesis and biological evaluation of omega-(N, N, N-trialkylammonium)alkyl esters and thioesters of carboxylic acid nonsteroidal antiinflammatory agents. Pharm Res.

[CR38] Vukasinovic-Milic T, Rakin M, Siler-Marinkovic S (2007). Utilization of baker's yeast (*Saccharomyces cerevisiae*) for the production of yeast extract: effects of different enzymatic treatments on solid, protein and carbohydrate recovery. J Serb Chem Soc.

[CR39] Walker C, Ryu S, Giannone RJ, Garcia S, Trinh CT (2020). Understanding and eliminating the detrimental effect of thiamine deficiency on the oleaginous yeast yarrowia lipolytica. Appl Environ Microbiol.

[CR40] Wu B, Wang X, Yang L, Yang H, Zeng H, Qiu YM, Wang CJ, Yu J, Li JP, Xu DH, He ZL, Chen SW (2016). Effects of *Bacillus amyloliquefaciens ZM9* on bacterial wilt and rhizosphere microbial communities of tobacco. Appl Soil Ecol.

[CR41] Yang MH, Xing JM (2017). Improvement of pyruvate production based on regulation of intracellular redox state in engineered *Escherichia coli*. Biotechnol Bioprocess Eng.

[CR42] Zeng W, Zhang H, Xu S, Fang F, Zhou J (2017). Biosynthesis of keto acids by fed-batch culture of *Yarrowia lipolytica* WSH-Z06. Bioresour Technol.

[CR43] Zhang C, Zhong CQ, Wu DJ (2021). Study on the reuse process of hydrolysate from gamma-polyglutamic acid fermentation residues. Arab J Chem.

[CR44] Zhou J, Huang L, Liu L, Chen J (2009). Enhancement of pyruvate productivity by inducible expression of a F(0)F(1)-ATPase inhibitor INH1 in *Torulopsis glabrata* CCTCC M202019. J Biotechnol.

[CR45] Zhou XH, Bao XH, Zhou J, Xin FX, Zhang WM, Qian XJ, Dong WL, Jiang M, Ochsenreither K (2021). Evaluating the effect of cultivation conditions on palmitoleic acid-rich lipid production by *Scheffersomyces segobiensis DSM* 27193. Biofuels Bioproducts & Biorefining-Biofpr.

[CR46] Zotkina VP, Koroleva VA (1984). Clinico-physiological characteristics of the contact action of high-frequency ultrasound. Gig Sanit.

