# Supplementary material for: Production of pyruvic acid with Candida glabrata using self-fermenting spent yeast cell dry powder as a seed nitrogen source
Source: Bioresour Bioprocess. 2022 Oct 17;9(1):109. doi: 10.1186/s40643-022-00593-5 (PMC10991669; doi:10.1186/s40643-022-00593-5)
Supplement: Supplementary file 1 — Additional file 1: Figure S1. Detection the remaining amounts of four vitamins. Table S1. The remaining amounts of four vitamins at the end of fermentation [file 40643_2022_593_MOESM1_ESM.docx]

**Supplementary information**

**Production of pyruvic acid with *Candida glabrata* using self-fermenting spent yeast cell dry powder as a seed nitrogen source**

Qiyuan Lu^1,2,3,4^, Xiaoyu Shan^1,2,3,4^, Weizhu Zeng^1,2,3*^, Jingwen Zhou^1,2,3,4*^

^1^ Science Center for Future Foods, Jiangnan University, 1800 Lihu Road, Wuxi, Jiangsu 214122, China.

^2^ National Engineering Laboratory for Cereal Fermentation Technology, Jiangnan University, 1800 Lihu Road, Wuxi, Jiangsu 214122, China.

^3^ School of Biotechnology and Key Laboratory of Industrial Biotechnology, Ministry of Education, Jiangnan University, 1800 Lihu Road, Wuxi, Jiangsu 214122, China;

^4^ Jiangsu Provisional Research Center for Bioactive Product Processing Technology, Jiangnan University, 1800 Lihu Road, Wuxi, Jiangsu 214122, China;

* Corresponding author: Weizhu Zeng, Jingwen Zhou

Mailing address: Science Center for Future Foods, Jiangnan University, 1800 Lihu Road, Wuxi, Jiangsu 214122, China

Phone: +86-510-85914371, Fax: +86-510-85914371

E-mail: zwzeng@jiangnan.edu.cn, zhoujw1982@jiangnan.edu.cn.


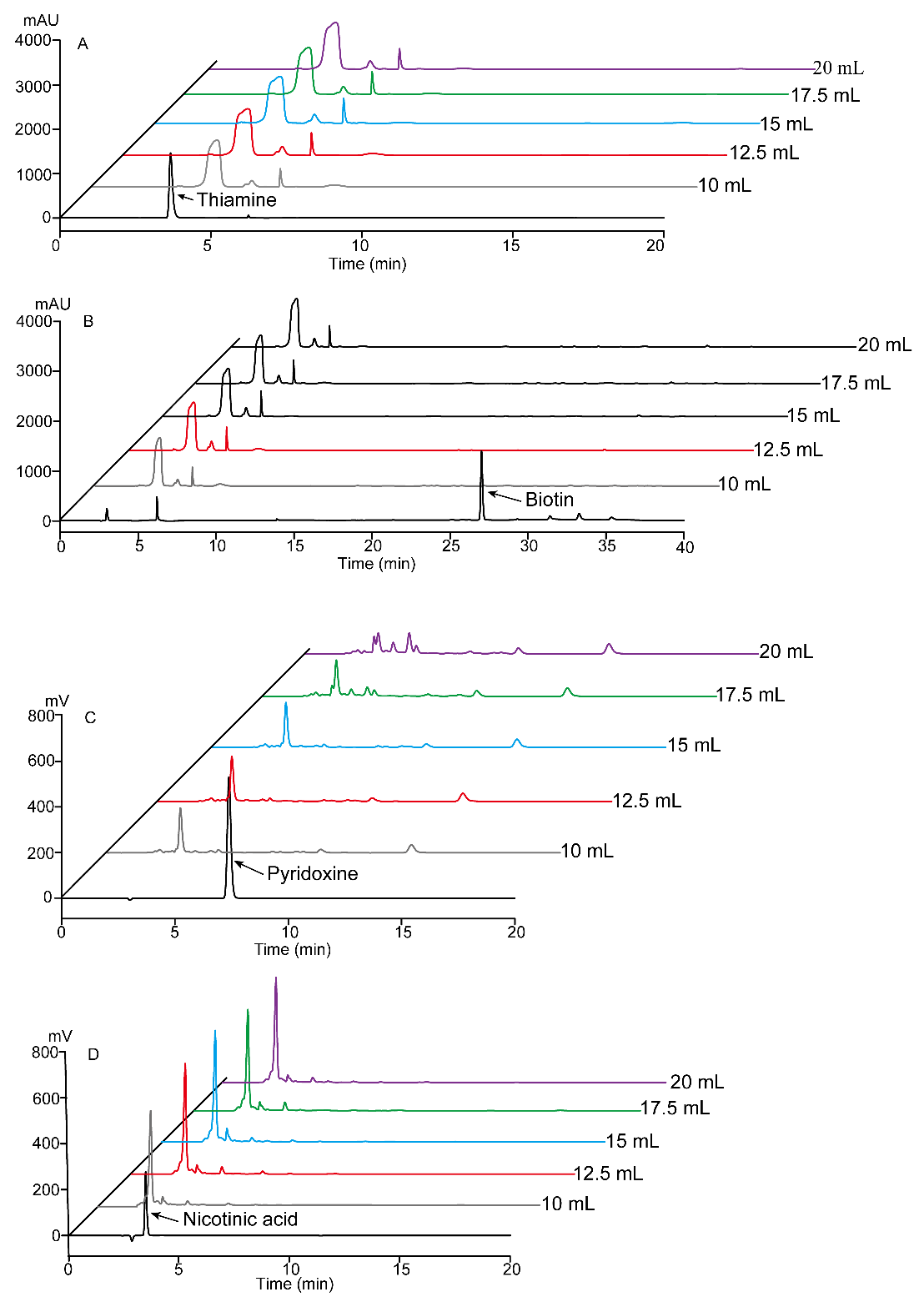


**Figure S1. Detection the remaining amounts of four vitamins**

A: The remaining amounts of different concentrations of thiamine. B: The remaining amounts of different concentrations of biotin. C: The remaining amounts of different concentrations of pyridoxine. D: The remaining amounts of different concentrations of nicotinic acid.

**Table S1. the remaining amounts of four vitamins at the end of fermentation**

| Addition  (mL/L) | Thiamine (mg/L) | Biotin  (mg/L) | Pyridoxine  (mg/L) | Nicotinic acid (mg/L) |
| --- | --- | --- | --- | --- |
| 10.0 | N.A | N.A | N.A | N.A |
| 12.5 | N.A | N.A | N.A | N.A |
| 15.0 | N.A | N.A | N.A | N.A |
| 17.5 | N.A | N.A | N.A | 23.60 |
| 20.0 | N.A | N.A | N.A | 41.33 |

N.A indicates that the data are unavailable due to a low concentration of the target compound that exceeds the HPLC detection limit or the absence of the target chemical in the sample.
